# A Japanese case of mitochondrial 3‐hydroxy‐3‐methylglutaryl‐CoA synthase deficiency who presented with severe metabolic acidosis and fatty liver without hypoglycemia

**DOI:** 10.1002/jmd2.12051

**Published:** 2019-06-03

**Authors:** Tomoko Lee, Yuichi Takami, Kenji Yamada, Hironori Kobayashi, Yuki Hasegawa, Hideo Sasai, Hiroki Otsuka, Yasuhiro Takeshima, Toshiyuki Fukao

**Affiliations:** ^1^ Department of Pediatrics Hyogo College of Medicine Nishinomiya Japan; ^2^ Department of Pediatrics Japanese Red Cross Society Himeji Hospital Himeji Japan; ^3^ Department of Pediatrics Shimane University Faculty of Medicine Izumo Japan; ^4^ Department of Pediatrics Graduate School of Medicine, Gifu University Gifu Japan

**Keywords:** fatty liver, glutarate, HMG‐CoA synthase deficiency, ketogenesis

## Abstract

Mitochondrial 3‐hydroxy‐3‐methylglutaryl‐CoA synthase deficiency (mHS deficiency) is a rare autosomal recessive inborn error of ketogenesis caused by a mutation in the *HMGCS2* gene, which is characterized by non‐(hypo)‐ketotic hypoglycemia, lethargy, and hepatomegaly during acute infection and/or prolonged fasting. Clinical presentations are similar to fatty acid oxidation defects; however, diagnosis of mHS deficiency is difficult because of poor biochemical markers. We report the case of a 12‐month‐old Japanese boy with mHS deficiency who presented with a coma, and hepatomegaly, but no hypoglycemia after a febrile episode and poor oral intake. Metabolic acidosis and severe fatty liver were observed. Serum acylcarnitine analysis revealed a slightly decreased free carnitine (C0) level and an increased acetylcarnitine (C2) level. Urinary organic acid analysis revealed hypoketotic dicarboxylic aciduria, and increased excretions of glutarate, and, retrospectively, 4‐hydroxy‐6‐methyl‐2‐pyrone. Although the patient did not present with hypoglycemia, the severe fatty liver and elevated free fatty acids to total ketone bodies ratio strongly suggested an inborn error of ketogenesis. In the analysis of the *HMGCS2* gene, compound heterozygous mutations of c.130_131ins C (L44PfsX29) and c.1156_1157insC (L386PfsX73) were identified, which led to the diagnosis of mHS deficiency. He had recovered without any complication by the therapy, including intravenous glucose infusion. Unlike the previously reported cases of mHS deficiency, our case did not present with hypoglycemia and the fatty liver lasted over several months. mHS deficiency should be taken into consideration when a patient has severe metabolic acidosis and fatty liver with no or subtle ketosis, even without hypoglycemia.

## 1 INTRODUCTION

Ketone bodies, generated from fatty acids, play an important role as an alternative energy source when glucose supply is low.^1^, ^2^ Mitochondrial 3‐hydroxy‐3‐methylglutaryl‐CoA (HMG‐CoA) synthase (EC2.3.3.10) is a key enzyme that mediates the rate‐limiting step of ketone body synthesis, catalyzing the condensation reaction between the acetyl‐CoA and acetoacetyl‐CoA to HMG‐CoA.^3^

Mitochondrial HMG‐CoA synthase deficiency (mHS deficiency; OMIM No. 605991) is a rare autosomal recessive inborn error of ketogenesis caused by mutations in the *HMGCS2* (3‐hydroxy‐3‐methylglutaryl‐CoA synthase‐2) gene, located on chromosome 1p12‐13, which consists of 10 exons.^4^, ^5^, ^6^

mHS deficiency was first described in 1997.^7^ To date, more than 20 cases have been reported.^8^, ^9^, ^10^, ^11^, ^12^, ^13^, ^14^, ^15^, ^16^, ^17^ Most patients presented with hypoglycemia and hepatomegaly during acute infection and prolonged fasting and showed an absence of clinical symptoms in the intermittent phase. Non‐(hypo)‐ketotic hypoglycemia with high free fatty acids (FFA) is the predominant laboratory finding.^18^ Clinical presentations are similar to fatty acid β‐oxidation defects, however, no specific acylcarnitine profile is found in mHS deficiency. Although 4‐hydroxy‐6‐methyl‐2‐pyrone (4HMP) in urine was recently reported as a possible useful specific marker,^13^ diagnosis of mHS deficiency is still challenging owing to poor biochemical markers. Therefore, diagnosis of mHS deficiency is based on genetic analysis.

Here, we report the case of a Japanese boy with mHS deficiency who did not present with hypoglycemia in the acute phase. We describe the detailed clinical course and characteristics of the present case together with previously reported cases.

## 2 CASE PRESENTATION

A 12‐month‐old Japanese boy born to nonconsanguineous parents was a second child with a healthy elder sister. He had grown and developed normally. No abnormalities were detected in a newborn screenings using tandem mass spectrometry. He presented with a fever, upper respiratory symptoms, and loss of oral intake. After 7 days of febrile illness, he was admitted to the hospital because of polypnea and cyanosis. Influenza A was detected in a rapid test. Laboratory investigation (Supporting Information Table S1) revealed mild hyperammonemia (173 μg/dL), elevated aspartate aminotransferase (AST; 461 IU/L) and alanine aminotransferase (ALT; 142 IU/L) levels and severe metabolic acidosis (pH 6.985, pCO_2_ 13.8 mmHg, HCO_3_ 3.2 mmol/L, BE −26.5 mmol/L; and anion gap, 30.5 mmol/L). The creatine kinase, lactic acid, and pyruvic acid levels were normal. Hypoglycemia was not detected (9.39 mmol/L) before glucose infusion was started. While the level of FFA was elevated (1.31 mmol/L), that of acetoacetic acid did not increase (0.04 mmol/L) and that of 3‐hydroxybutyrate was only mildly elevated (0.154 mmol/L).

**Figure 1:**
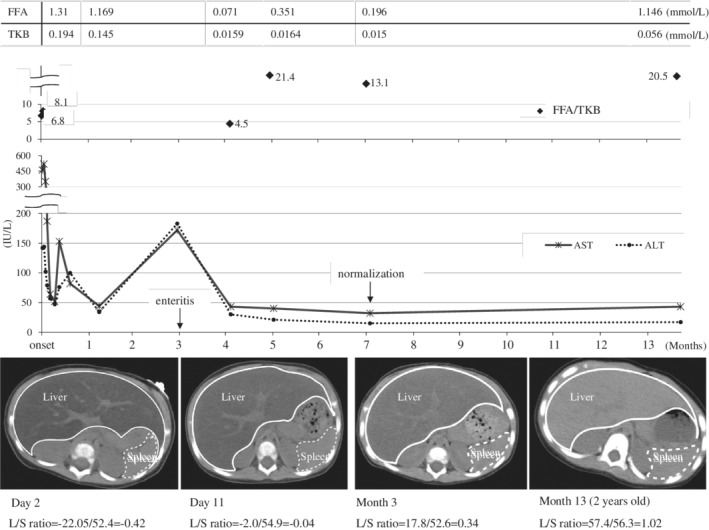
The clinical course and laboratory data. The ratio of FFA to total ketone bodies (6.75; normal range, 0.3‐2.5) was high at onset and even much higher in the intermittent phase. The AST and ALT values were extremely elevated at onset and then decreased dramatically. Around 3 months after the first crisis, the values spiked due to enteritis. In 7 months after the onset, the AST and ALT values finally normalized. The CT scan performed on day 2 revealed a prominent, enlarged and fatty liver. On day 11, these findings had improved somewhat. In month 3, the liver had enlarged again due to enteritis. In 13 months, the fatty liver had mostly disappeared, but the hepatomegaly still remained. *L/*S* ratio: the ratio of the CT value in the liver to that in the spleen. An *L*/*S* ratio of <1.0 mean fatty liver. ALT, alanine aminotransferase; AST, aspartate aminotransferase; CT, computed tomography; FFA, free fatty acids

Physical examination revealed marked hepatomegaly. Abdominal computed tomography (CT) revealed severe fatty liver and hepatomegaly without splenomegaly (Figure 1).

After admission, the patient had a convulsion, which was immediately treated with diazepam (1 mg/kg). However, he did not regain consciousness and had no reaction to pain. Intravenous infusion including glucose was initiated after taking critical blood samples. Peramivir was also administered against the influenza A infection. Administration of carnitine and vitamin cocktail therapy including vitamin B1, B2, B12, C, and biotin was started, considering the possibility of mitochondrial dysfunction. At the second day of admission, his consciousness did not improve, and the severe metabolic acidosis lasted even after correction with sodium bicarbonate. Continuous electroencephalography monitoring revealed no high‐amplitude slow wave. On the third day, his symptoms improved dramatically. The patient reacted to stimulation and could open his eyes. He could sit by himself on day 4, and his consciousness became completely normal on day 5. The fatty liver and hepatomegaly were improved somewhat on day 11 (Figure 1). A brain MRI performed on day 10 revealed no abnormality. The patient was discharged without any complication on day 14.

After discharge, he was managed with a normal diet and avoidance of long fasting periods. Around 3 months after the onset, he had enteritis. He overcame it without metabolic crisis with intravenous glucose administration at the local hospital. However, his liver was enlarged and his AST and ALT levels spiked again (Figure 1). In 7 months after the onset, his AST and ALT levels finally normalized. In 13 months after the onset, the fatty liver had mostly disappeared, but mild hepatomegaly was still detected on CT. After the first episode of metabolic crisis, the patient experienced no further episodes of metabolic crisis even with minor illnesses. At the age of 6 years, he had grown and developed normally.

## 3 MATERIALS AND METHODS

### 3.1 Biochemical analysis

Urinary organic acid levels were analyzed using gas chromatography mass spectrometry (GC/MS; QP‐2010 plus; Shimazu, Kyoto, Japan) at Shimane University, Japan, after solvent extraction and oxime‐trimethylsilyl derivatization of urine samples as previously described.^19^, ^20^ Urine metabolites were semi‐quantified by calculation of the relative peak area ratio (RPA, %) of each compound to margaric acid as the internal standard.^20^

The serum acylcarnitine level was analyzed using tandem mass spectrometry (MS/MS) (API‐3000; Applied Biosystems, Foster City, California) at Shimane University, Japan, after butyl‐derivatization of samples, as previously described.^21^, ^22^

### 3.2 Mutation analysis

Genomic DNA from the peripheral blood was extracted using standard phenol‐chloroform extraction methods. Mutation analysis was performed at the genomic level using polymerase chain reaction and direct sequencing, using a set of primer pairs that amplify fragments, including whole coding regions in all 10 exons and part of the 3′ flanking regions of *HMGCS2*. The primer sequences are presented in Supporting Information Table S2. The genomic *HMGCS2* sequence was obtained from the NCBI Reference Sequence Database (accession No. NG_013348.1/NM_005518.3).

## 4 RESULTS

Urinary organic acid obtained from the patient on admission was analyzed (Supporting Information Figure S1). While the levels of dicarboxylic acids (glutarate, adipate, suberate, octenediate, 3‐OH‐adipate, 3‐OH‐suberate, and 3‐OH‐sebacate) were markedly increased, the excretion of ketone bodies (3‐OH‐butyrate and acetoacetate) was only mildly elevated. The increased excretion of glutarate was prominent (823.77%, normal range; <4.00%). No other significant disease‐specific findings were detected at that time. 4HMP, which was retrospectively detected after the report of Pitt et al,^13^ was not detected at the time of diagnosis.

Serum acylcarnitine analysis revealed slightly decreased free carnitine (C0) level (12.56 μM; normal range, 20‐60 μM) and increased acetylcarnitine level (C2; 92.1 μM; normal range, 4‐60 μM) (Supporting Information Table S3). The levels of several kinds of acylcarnitine were slightly elevated, which were considered nonspecific reactions to the catabolic condition. After administration of carnitine, the C2 level was reduced but was still elevated (63.19 μM), as reported in the previous report.^9^ These abnormal findings in the acute phase (during metabolic disruption) were no longer detected in the intermittent phase (between acute phases).

Fatty acid β‐oxidation defects were suggested from the clinical findings; however, the results of the biochemical analysis were not specific to fatty acid β‐oxidation defects. Therefore, we focused on the inborn errors of ketogenesis because the ratio of FFA to total ketone bodies (TKB) was markedly elevated (6.75; normal range for infants, 0.3‐2.5). The analysis of the *HMGCS2 gene* revealed two heterozygous mutations of c.130_131ins C (L44PfsX29) and c.1156_1157insC (L386PfsX73) (Supporting Information Figure S2). Both were novel frameshift null mutations considered as pathogenic. The mutation of c.130_131insC (L44PfsX29) was inherited from the mother. The other mutation of c.1156_1157insC (L386PfsX73), which was also found in the elder sister, was suggested to be inherited from his father; however, analysis for his father could not be performed. These results confirmed the diagnosis of mHS deficiency.

## 5 DISCUSSION

We report a case with mHS deficiency, which was the second case diagnosed in Japan (Fukao et al, unpublished data). The previous cases are summarized in Table 1. Similar to other cases, our case presented with coma, hepatomegaly, fatty liver, and severe metabolic acidosis during acute infection and poor intake. While all previous cases presented with hypoglycemia except one case, which was investigated after infusion supply,^11^ our case did not present with hypoglycemia. Our patient did not receive any glucose supply before blood examination on admission. The reason why hypoglycemia did not occur was unknown, but influenza virus infection might be related.

**Table 1:** Patients with mHS deficiency

Case	Onset	Hypoglycemia	Fatty liver/hepatomegaly	Acylcarnitine profiles (in the acute phase)	Organic acid profiles (in the acute phase)	HMGCS2 mutations	References
1	6 y	+	+	Normal carnitine No more information	Normal	F174L/F174L	7 10
2	1 y 4 m	+	+	Normal	Dicarboxylic aciduria	R424X/?	12
3	11 m	+	+	Normal	Dicarboxylic aciduria	G212R/R500H	8
4	9 m	+	+	Normal	Dicarboxylic aciduria	G212R/IVS5+1g>a	17
5	4.5 y	+	+	Normal	Dicarboxylic aciduria	V54M/Y167C	16
6^a^	19 m	No data	+	Normal	Dicarboxylic aciduria	V54M/Y167C	16
7	7 m	+	+	Low C0 Elevated C2 (dramatically increased after carnitine supplementation)	Dicarboxylic aciduria	R188H/M307T	9
8^b^	1 y	No data	+	Normal (nonacute)	Normal (nonacute)	R188H/M307T	9
9	15 m	+	+	Normal	Dicarboxylic aciduria	G388R/R424X	15
10^c^	8 m	+	+	No data	No data	del ex1/del ex1	13
11^c^	9 m	+	+	No data	No data	del ex1/del ex1	13
12^c^	6 m	+	+	No data	4HMP Dicarboxylic aciduria	del ex1/del ex1	13
13^c^	10 m	+	+	No data	No data	del ex1/ del ex1	13
14	3 y 1 m	+	−	No data	No data	L266S/I407T	13
15	6 m	+	+	No data	No data	G388R/G388R	13
16^d^	No episode	−	−	No data	No data	G388R/G388R	13
17	2 y 5 m	+	−	No data	No data	G169D/R505Q	13
18	?	+	No data	No data	No data	del ex1/del ex1	13
19	?	+	No data	No data	No data	G212R/V144fs	13
20	?	+	No data	No data	No data	Q283A/G232V	13
21	?	+	No data	No data	No data	W185R/Y503C	13
22	?	+	No data	No data	No data	S360P/S360P	13
23	?	+	No data	No data	No data	G168S/F174L	13
24	8 m	– (after infusion)	+	Low C0 Elevated C2 after carnitine supplementation	Dicarboxylic aciduria Increased glutarate Elevated 4HMP	K137X/M381V	11
25	3 m	+	−	Normal	Dicarboxylic aciduria	R112W/?	14
26	11 m	+	−	Normal free carnitine Increased acylcarnitine	4HMP Dicarboxylic aciduria Hydroxydicarboxylic aciduria	V144L/V144L	14
27	3 y	+	−	No data	Dicarboxylic aciduria	R505Q/R505Q	14
28	12 m	−	+	Low C0 Elevated C2	Dicarboxylic aciduria Increased glutarate 4HMP	L44PfsX29/L386PfsX73	This report

a Sibling of #5.

b Sibling of #7.

c Family members, variable hepatomegaly was noted in several patients.

d Sibling of #15.

Our case showed marked hepatomegaly and very severe fatty liver at the onset. This may be associated with prolonged ketogenic stresses (7‐day febrile condition), rather than an acute episode of a few days illness in most cases. The fatty liver remained for >3 months, and hepatomegaly was still found at 13 months after the first crisis (Figure 1), on the contrary to the fatty acid oxidation defects. When he had enteritis 3 months after the first crisis, he did not present with hypoglycemia, but his AST and ALT values and liver size worsened again. That condition might be pre‐crisis.

The elevated FFA/TKB ratio (6.75; normal range, 0.3‐2.5) in this case likely suggested an inborn errors of ketogenesis as described previously.^18^ The FFA/TKB ratio had been high after recovering from the metabolic episode (Figure 1). The values were higher in the intermittent phase than in the acute phase, maybe due to the increased ketogenesis from the branched chain amino acids in the acute phase.

The urine organic acid profile of our case was similar to those detected in previous cases. 4HMP was retrospectively detected in our case. As 4HMP has been reported to be a characteristic of mHS deficiency during hypoglycemic crisis,^13^ it is a possible specific biochemical marker to diagnose the disease. In addition, our case showed increased excretion of glutarate, which was only reported in another case.^11^ We also detected increased excretion of glutarate in the other cases of Japanese mHS deficiency (Fukao et al, unpublished data). As increased glutarate excretion is not evident in hypoketotic dicarboxylic aciduria in beta‐oxidation defects, this may also be a characteristic marker of mHS deficiency, although the etiology of increased glutarate is unknown.

Decreased free carnitine (C0) and increased acetylcarnitine (C2) levels in the acute phase were observed in our case, which was quite similar to one previous case reported by Aledo et al.^9^ Conboy et al described that the C2/C0 ratio in the acute phase could be an additional biochemical signature of mHS deficiency.^11^ The C2/C0 ratio in our case was also markedly elevated (7.33; 99th percentile, 1.83). As plenty of acetyl‐CoA is produced by beta‐oxidation but is not used for ketogenesis during ketogenic stresses such as fasting and febrile conditions in mHS deficiency, acetyl‐CoA should turn to acetylcarnitine in the liver to maintain the free CoA level. Hence, elevated C2 is expected in the catabolic condition in mHS‐deficient patients. Rapid increase in C2 level may result in C0 consumption. These findings could be the basis to suspect this disease. Furthermore, the fact that these abnormalities were only observed in the acute phase is also important.

Our patient had two frame‐shift mutations. To date, this is the only patient with two apparent null mutations beside homozygous deletion of exon 1.^13^ His phenotype was not more severe than those of other cases with missense mutations. At the age of 1 year, after 14 hours of fasting from the last meal, he did not have hypoglycemia. The fasting tolerance in this patient is considered to be longer than 14 hours, while it was 12‐19 hours in other cases.^7^, ^8^, ^12^, ^16^ He had not experienced any metabolic crisis until 6 years of age. The clinical findings in our case suggest no apparent genotype‐phenotype correlations in this disorder.

The onset of previous cases was around the age of 0‐6 years (Table 1). To date, no episode of metabolic decompensation has been reported after the age of 7 years. Despite the severity of metabolic compensation, most cases had good prognosis. However, some cases were reported to have retardation of cognitive and physical development or died from metabolic compensation.^11^, ^18^ Proper treatment during the onset of the sickness and in the acute crisis in patients with mHS deficiency is crucial. Therefore, the diagnosis at the first episode of metabolic compensation is important.

Here, we report some additional biochemical characteristics such as increased glutamate in urine and decreased free carnitine (C0) and increased acetylcarnitine (C2) levels in blood during acute crisis. Furthermore, we reported the elevated FFA/TKB ratio not only during crisis but also in the intermittent phase, which can be a diagnostic indicator. In addition, mHS deficiency cannot be excluded in a patient with severe metabolic acidosis with no or subtle ketosis and fatty liver, even without hypoglycemia.

### ONE SENTENCE TAKE‐HOME MESSAGE

Mitochondrial 3‐hydroxy‐3‐methylglutaryl‐CoA (HMG‐CoA) synthase deficiency (mHS deficiency) should be taken into consideration when a patient has severe metabolic acidosis and fatty liver with no or subtle ketosis, even without hypoglycemia.

### DETAILS OF THE CONTRIBUTIONS OF INDIVIDUAL AUTHORS

Tomoko Lee, Yuichi Takami, and Yasuhiro Takeshima were involved in clinical management of the patient. Kenji Yamada, Hironori Kobayashi, and Yuki Hasegawa performed biochemical analyses including urinary organic acid analysis and acylcarnitine analysis. Hideo Sasai, Hiroki Otsuka, and Toshiyuki Fukao performed genetic analysis. Tomoko Lee, Yasuhiro Takeshima, and Toshiyuki Fukao planned this study. Tomoko Lee wrote the first draft. Toshiyuki Fukao intensively reviewed and revised the manuscript.

### COMPETING INTEREST STATEMENT

Tomoko Lee, Yuichi Takami, Kenji Yamada, Hironori Kobayashi, Yuki Hasegawa, Hideo Sasai, Hiroki Otsuka, Yasuhiro Takeshima, and Toshiyuki Fukao declare that they have no conflict of interest.

### DETAILS OF ETHICS APPROVAL

This study was approved by the Ethics Committee of Hyogo College of Medicine, Japan and the Graduate School of Medicine, Gifu University, Japan.

### PATIENT CONSENT STATEMENT

All procedures followed were in accordance with the ethical standards of the responsible committee on human experimentation (institutional and national) and with the Helsinki Declaration of 1975, as revised in 2000. Informed consent was obtained from the patients' mother for being included in the study.

### DOCUMENTATION OF APPROVAL FROM THE INSTITUTIONAL COMMITTEE FOR CARE AND USE OF LABORATORY ANIMALS

Not applicable. This article does not contain any studies with animal subjects performed by the any of the authors.

## Supporting information


**Figure S1.** The profiles of urinary organic acid analysis using GC/MS. A, In the acute phase; B, In the intermittent phase

**Figure S2.** Sequence analysis of the *HMGCS2* gene. Two heterozygous mutations of c.130_131ins C (L44PfsX29) and c.1156_1157insC (L386PfsX73) were identified

**Table S1.** Laboratory examination on admission (before infusion glucose)

**Table S2.** Amplification primer sets for *HMGCS2* exons

**Table S3.** The acylcarnitine profiles of serum samples in the acute phase
