# Supplementary material for: A Japanese case of mitochondrial 3‐hydroxy‐3‐methylglutaryl‐CoA synthase deficiency who presented with severe metabolic acidosis and fatty liver without hypoglycemia
Source: JIMD Rep. 2019 Jun 3;48(1):19–25. doi: 10.1002/jmd2.12051 (PMC6606983; doi:10.1002/jmd2.12051)
Supplement: Supplementary file 1 — Figure S1. The profiles of urinary organic acid analysis using GC/MS. A, In the acute phase; B, In the intermittent phase [file JMD2-48-19-s001.pptx]

## Slide 1
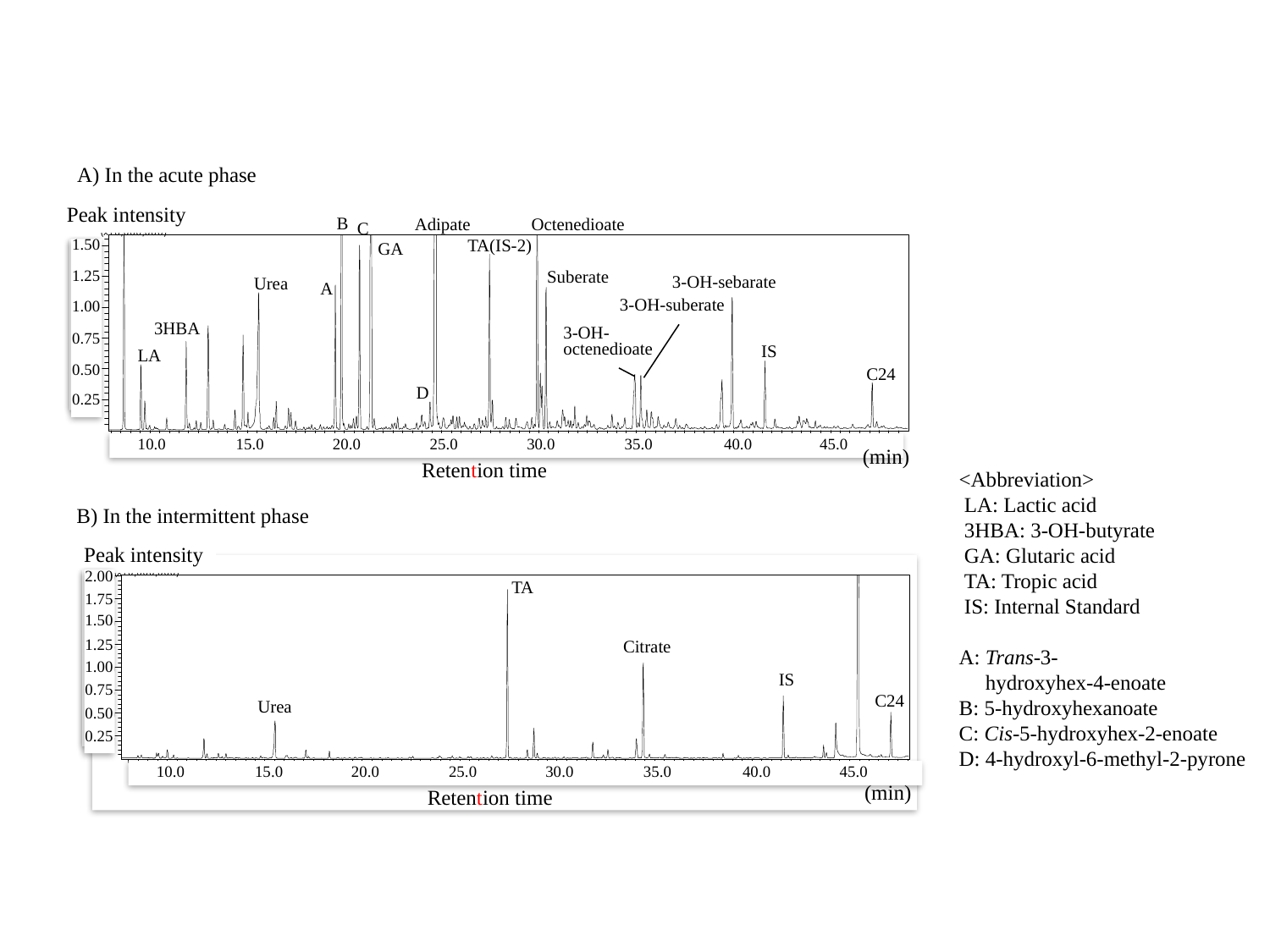

A) In the acute phase
Peak intensity
B
Adipate
Octenedioate
C
1.50
TA(IS-2)
GA
Suberate
1.25
3-OH-sebarate
Urea
A
3-OH-suberate
1.00
3HBA
3-OH-
octenedioate
0.75
IS
LA
0.50
C24
D
0.25
10.0
15.0
20.0
25.0
30.0
35.0
40.0
45.0
(min)
Retention time
<Abbreviation>
 LA: Lactic acid
 3HBA: 3-OH-butyrate
 GA: Glutaric acid
 TA: Tropic acid
 IS: Internal Standard
A: Trans-3-
 hydroxyhex-4-enoate
B: 5-hydroxyhexanoate
C: Cis-5-hydroxyhex-2-enoate
D: 4-hydroxyl-6-methyl-2-pyrone
B) In the intermittent phase
Peak intensity
TA
Citrate
IS
C24
Urea
(min)
Retention time
2.00
1.75
1.50
1.25
1.00
0.75
0.50
0.25
10.0
15.0
20.0
25.0
30.0
35.0
40.0
45.0
