# Supplementary material for: A Japanese case of mitochondrial 3‐hydroxy‐3‐methylglutaryl‐CoA synthase deficiency who presented with severe metabolic acidosis and fatty liver without hypoglycemia
Source: JIMD Rep. 2019 Jun 3;48(1):19–25. doi: 10.1002/jmd2.12051 (PMC6606983; doi:10.1002/jmd2.12051)
Supplement: Supplementary file 2 — Figure S2. Sequence analysis of the HMGCS2 gene. Two heterozygous mutations of c.130_131ins C (L44PfsX29) and c.1156_1157insC (L386PfsX73) were identified [file JMD2-48-19-s002.pptx]

## Slide 1
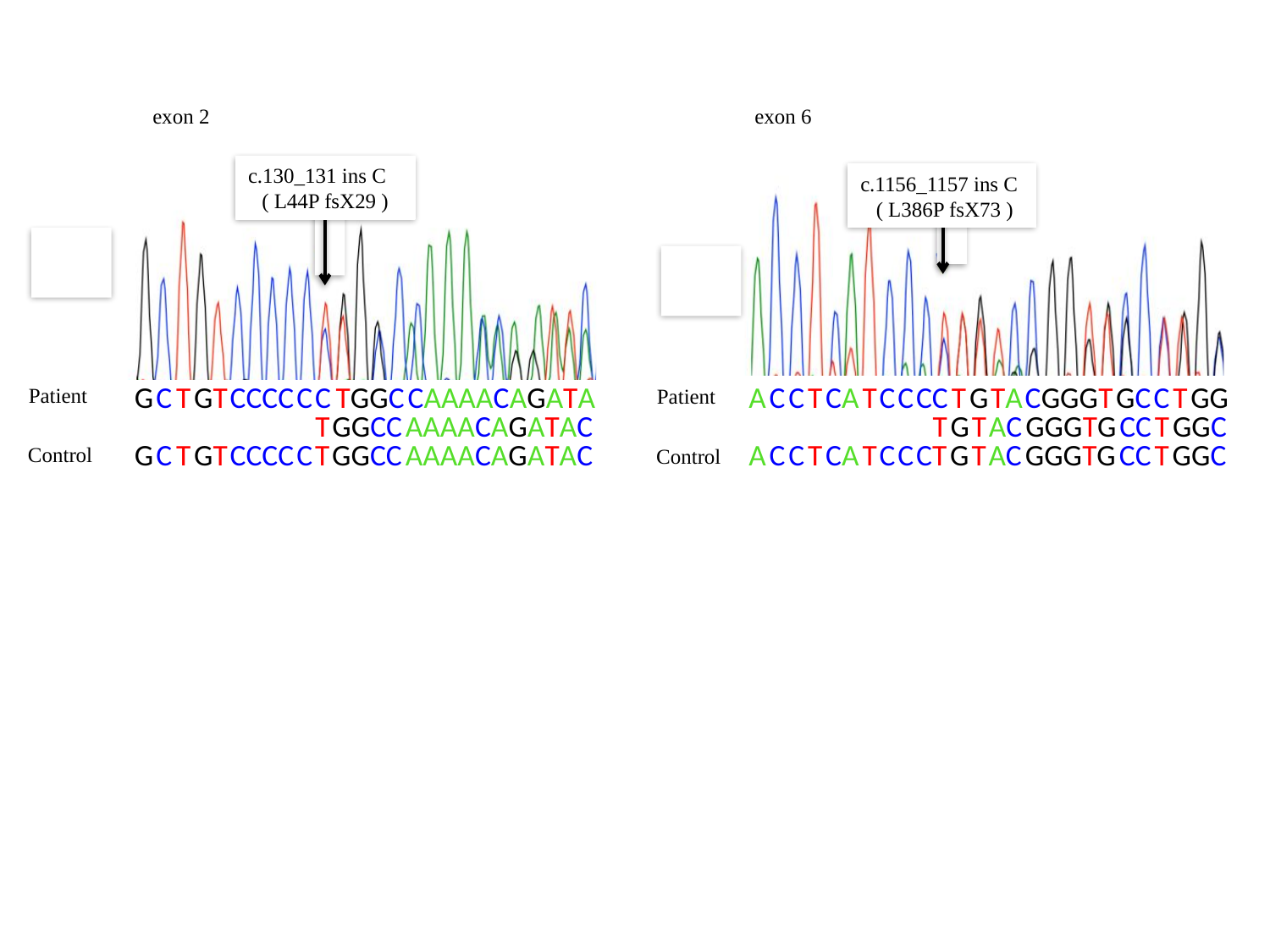

exon 2
exon 6
c.130_131 ins C
( L44P fsX29 )
c.1156_1157 ins C
 ( L386P fsX73 )
v
v
Patient
Patient
G C T GT CCCC C C TGGC CAAAACAGATA
 T GGCC AAAACAGATAC
G C T GT CCCC C T GGCC AAAACAGATAC
A C C T CA T C C CC T G TA CGGGT GC C T GG
 T G T AC GGGTG CC T GGC
A C C T CA T C C CT G T AC GGGTG CC T GGC
Control
Control
