# Supplementary material for: A Japanese case of mitochondrial 3‐hydroxy‐3‐methylglutaryl‐CoA synthase deficiency who presented with severe metabolic acidosis and fatty liver without hypoglycemia
Source: JIMD Rep. 2019 Jun 3;48(1):19–25. doi: 10.1002/jmd2.12051 (PMC6606983; doi:10.1002/jmd2.12051)
Supplement: Supplementary file 3 — Table S1. Laboratory examination on admission (before infusion glucose) [file JMD2-48-19-s003.pptx]

## Slide 1
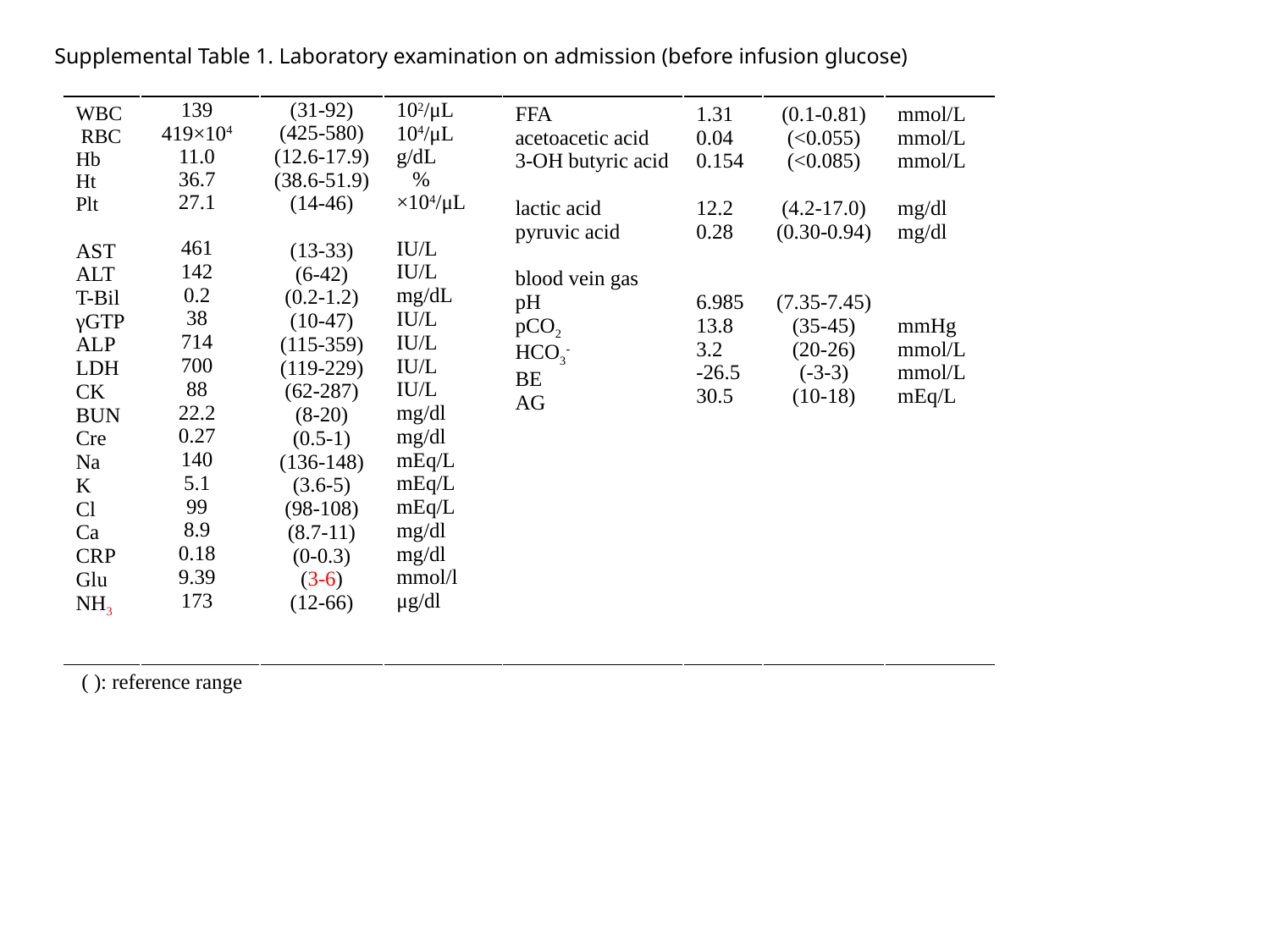

Supplemental Table 1. Laboratory examination on admission (before infusion glucose)
| WBC RBC Hb Ht Plt AST ALT T-Bil γGTP ALP LDH CK BUN Cre Na K Cl Ca CRP Glu NH3 | 139 419×104 11.0 36.7 27.1 461 142 0.2 38 714 700 88 22.2 0.27 140 5.1 99 8.9 0.18 9.39 173 | (31-92) (425-580) (12.6-17.9) (38.6-51.9) (14-46) (13-33) (6-42) (0.2-1.2) (10-47) (115-359) (119-229) (62-287) (8-20) (0.5-1) (136-148) (3.6-5) (98-108) (8.7-11) (0-0.3) (3-6) (12-66) | 102/μL 104/μL g/dL % ×104/μL IU/L IU/L mg/dL IU/L IU/L IU/L IU/L mg/dl mg/dl mEq/L mEq/L mEq/L mg/dl mg/dl mmol/l μg/dl | FFA acetoacetic acid 3-OH butyric acid lactic acid pyruvic acid blood vein gas pH pCO2 HCO3- BE AG | 1.31 0.04 0.154 12.2 0.28 6.985 13.8 3.2 -26.5 30.5 | (0.1-0.81) (<0.055) (<0.085) (4.2-17.0) (0.30-0.94) (7.35-7.45) (35-45) (20-26) (-3-3) (10-18) | mmol/L mmol/L mmol/L mg/dl mg/dl mmHg mmol/L mmol/L mEq/L |
| --- | --- | --- | --- | --- | --- | --- | --- |
( ): reference range
