# Supplementary material for: A Japanese case of mitochondrial 3‐hydroxy‐3‐methylglutaryl‐CoA synthase deficiency who presented with severe metabolic acidosis and fatty liver without hypoglycemia
Source: JIMD Rep. 2019 Jun 3;48(1):19–25. doi: 10.1002/jmd2.12051 (PMC6606983; doi:10.1002/jmd2.12051)
Supplement: Supplementary file 4 — Table S2. Amplification primer sets for HMGCS2 exons [file JMD2-48-19-s004.pptx]

## Slide 1
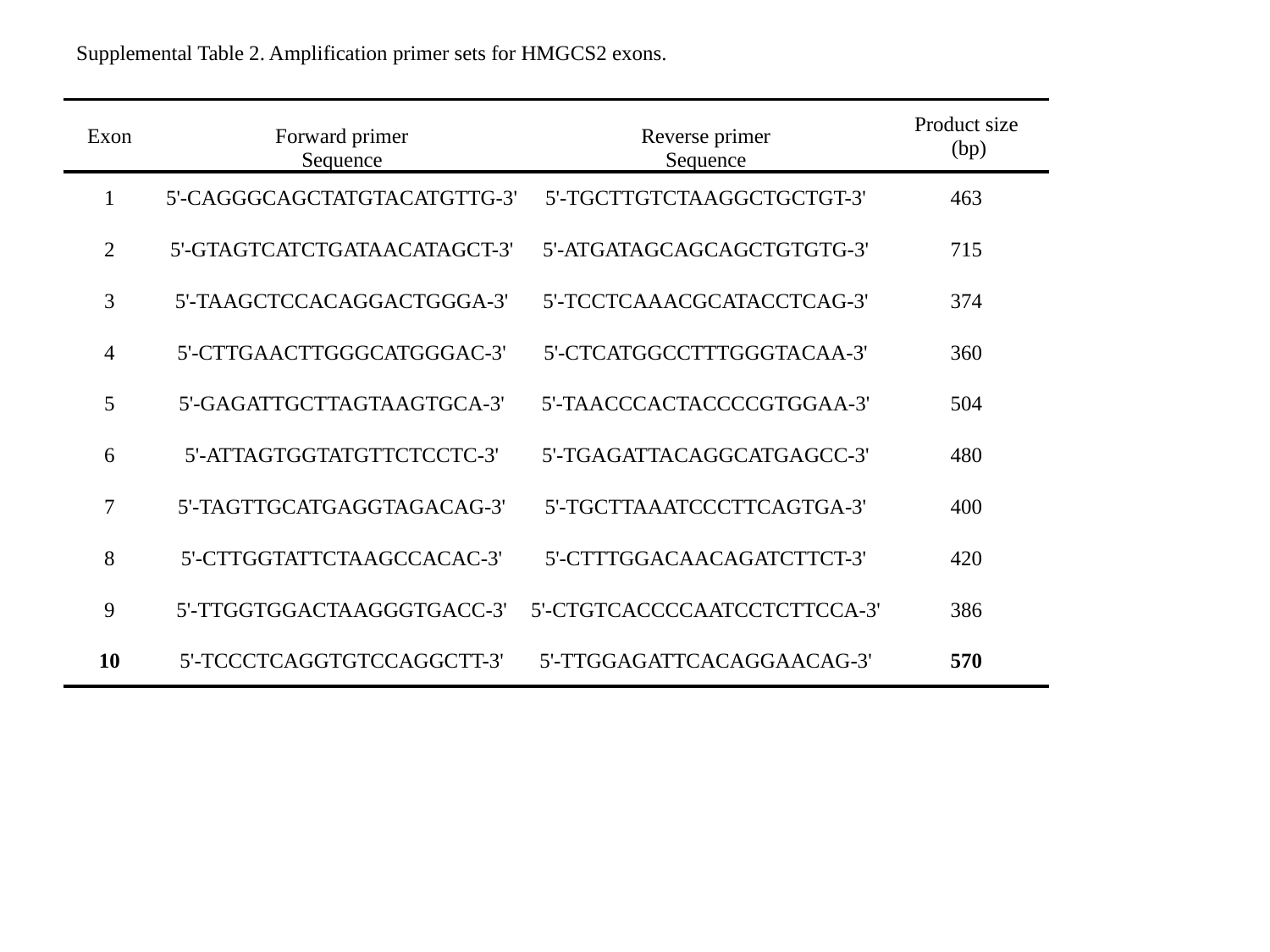

Supplemental Table 2. Amplification primer sets for HMGCS2 exons.
| Exon | Forward primer Sequence | Reverse primer Sequence | Product size (bp) |
| --- | --- | --- | --- |
| 1 | 5'-CAGGGCAGCTATGTACATGTTG-3' | 5'-TGCTTGTCTAAGGCTGCTGT-3' | 463 |
| 2 | 5'-GTAGTCATCTGATAACATAGCT-3' | 5'-ATGATAGCAGCAGCTGTGTG-3' | 715 |
| 3 | 5'-TAAGCTCCACAGGACTGGGA-3' | 5'-TCCTCAAACGCATACCTCAG-3' | 374 |
| 4 | 5'-CTTGAACTTGGGCATGGGAC-3' | 5'-CTCATGGCCTTTGGGTACAA-3' | 360 |
| 5 | 5'-GAGATTGCTTAGTAAGTGCA-3' | 5'-TAACCCACTACCCCGTGGAA-3' | 504 |
| 6 | 5'-ATTAGTGGTATGTTCTCCTC-3' | 5'-TGAGATTACAGGCATGAGCC-3' | 480 |
| 7 | 5'-TAGTTGCATGAGGTAGACAG-3' | 5'-TGCTTAAATCCCTTCAGTGA-3' | 400 |
| 8 | 5'-CTTGGTATTCTAAGCCACAC-3' | 5'-CTTTGGACAACAGATCTTCT-3' | 420 |
| 9 | 5'-TTGGTGGACTAAGGGTGACC-3' | 5'-CTGTCACCCCAATCCTCTTCCA-3' | 386 |
| 10 | 5'-TCCCTCAGGTGTCCAGGCTT-3' | 5'-TTGGAGATTCACAGGAACAG-3' | 570 |
