# Supplementary material for: A Japanese case of mitochondrial 3‐hydroxy‐3‐methylglutaryl‐CoA synthase deficiency who presented with severe metabolic acidosis and fatty liver without hypoglycemia
Source: JIMD Rep. 2019 Jun 3;48(1):19–25. doi: 10.1002/jmd2.12051 (PMC6606983; doi:10.1002/jmd2.12051)
Supplement: Supplementary file 5 — Table S3. The acylcarnitine profiles of serum samples in the acute phase [file JMD2-48-19-s005.docx]

Supplemental Table 3 The acylcarnitine profiles of serum samples in the acute phase

| Acylcarnitine  (μM) | Reference value | On admission |  |
| --- | --- | --- | --- |
| C0  C2  C3  C4  C4-OH  C5:1  C5  C5-OH  C5-DC/C10-OH  C6  C8:1  C8  C10:1  C10  C12:1  C14:1  C14-OH  C16  C16:1  C16-OH  C18  C18:1  C18:1-OH  (C16+C18:1)/C2 | (20-60)  (4-60)  (<3.5)  (<1.85)  (<1.4)  (<0.04)  (<1.0)  (<0.16)  (<0.25)  (<0.46)  (<1.0)  (<1.0)  (<0.35)  (<0.8)  (<0.2)  (<0.1)  (<0.1)  (<0.5)  (<0.1)  (<0.8)  (<0.3)  (<0.46)  (<0.7)  (<0.36) | 12.56  92.1  1.78  0.54  1.05  0.043  0.14  0.052  0.61  0.39  0.15  0.54  0.41  0.85  0.65  0.73  0.089  0.46  0.18  0.038  0.15  0.49  0.041  0.01 | Low  High  High  High  High  High  High  High  High  High |
